# Emerging Roles of Fc Receptor-Like 1 in Immunotherapy of Diffuse Large B-Cell Lymphoma

**Published:** 2025-10-22

**Authors:** Kayce Blumenstock, Vandana Zaman, Camille Green, Narendra L. Banik, Azizul Haque

**Affiliations:** 1 Department of Pharmacology and Immunology, Medical University of South Carolina, 173 Ashley Avenue, Charleston, SC 29425, USA; 2 Ralph H. Johnson Veterans Administration Medical Center, 109 Bee St, Charleston, SC 29401, USA; 3 Department of Neurosurgery, Medical University of South Carolina, 96 Jonathan Lucas Street, Charleston, SC 29425, USA

**Keywords:** Fc receptor-like 1 (FCRL1), non-Hodgkin’s lymphoma (NHL), diffuse large B-cell lymphoma (DLBCL), immunoreceptor tyrosine-based activation motif (ITAM), immunoreceptor tyrosine-based inhibiting motif (ITIM), immunotherapy

## Abstract

Fc Receptor-Like 1 (FCRL1), a member of the FCRL family, contains two immunoreceptor tyrosine-based activation motifs (ITAMs) in its cytoplasmic domain and plays a critical role in B-cell biology. Its expression begins in pre-B-cells, dynamically shifts during B-cell development, and contributes to the regulation of human B-cell activation. Notably, FCRL1 is overexpressed in subsets of naive and memory B-cells, as well as in malignant B-cells, including those in diffuse large B-cell lymphoma (DLBCL), an aggressive and often treatment-resistant hematological malignancy. Among FCRL family members, FCRL1 stands out as a promising immunotherapeutic target due to its selective expression in malignant B-cells and its functional role in proliferation. Given the limited efficacy of current therapies for relapsed/refractory DLBCL, targeting FCRL1 could address an unmet clinical need by offering a novel, mechanism-based approach to modulate B-cell signaling and enhance anti-tumor immunity. This mini-review highlights the therapeutic potential of FCRL1-directed strategies, supporting their further exploration in preclinical models and future clinical trials for DLBCL and other B-cell malignancies.

## 1 Introduction

Antigen (Ag) recognition of tumor cells is essential for host defense, as well as potentiating the destruction and clearance of malignant cells. However, lymphoid malignancies such as lymphoma and leukemia often have insufficient anti-tumor immune responses [1–3]. The transformation of these cells compromises host defense, and many of these malignant cells evolve mechanisms to escape immune recognition [4,5]. Specifically, B-cell tumors vary greatly in how they behave in the body as well as in their clinical presentation [6]. While leukemic cells typically involve the bone marrow and peripheral blood, B-cell lymphomas usually produce masses in the lymph nodes or other tissues. Plasma cell tumors differ because they typically originate within the bones and can generate systemic symptoms due to the production of antibodies or immunoglobulins [7,8]. In any case, all of these neoplasms have the capacity to spread to various other tissues within the body. While current treatments such as chemotherapy and/or radiation are typically effective, they are largely non-specific and can cause damage to the healthy tissues that surround the targeted cancer cells.

Antibody-based immunotherapy is gaining momentum in treating cancer, but its effectiveness varies among cancer types and patients. Targeted immunotherapies have significantly advanced the treatment of B-cell malignancies, particularly through the use of monoclonal antibody treatments and chimeric antigen receptor T-cell (CAR-T) therapies [9,10]. CAR-T cells have shown remarkable success in killing malignant cells and specifically treating B-cell malignancies because they are able to target specific antigens [11], although antigen escape and toxicities remain a problem. CAR-T cells are a great example of the importance of identifying reliable tumor-associated antigens that can be used for treatment.

Because of the importance of finding biomarkers for prognosis, as well as possible therapeutic targets, for diffuse large B-cell lymphoma (DLBCL) and other B-cell malignancies, Fc receptor-like 1 (FCRL1) is being explored as a potential target. The interaction between antibodies and Fc receptors (FcRs) on immune cells is a critical factor in determining the activity of antibodies in the body. FCRL proteins have been seen almost exclusively in B-cells, including malignant B-cells. FCRL1 is distinct within its family due to the fact that it contains two immunoreceptor tyrosine-based activating motifs (ITAMs) and is highly expressed in several non-Hodgkin’s B-cell lymphomas. However, little is known about the functioning of these proteins in humans, so more exploration is needed. The FCRL isoform also appears to inhibit recognition of CD4^+^ T cells, and consequently would hinder T cell immunosurveillance of B-cell lymphoma, which may lead to unchecked growth of lymphoma. Studies suggest targeted antibody immunotherapy could be a first-line treatment strategy for B-cell lymphoma due to the success of rituximab, a chimeric antibody that binds to and eliminates cells expressing the pan-B cell marker CD20 [12,13]. Since FCRL isoforms also regulate signals to B-cells after ligation, antibody targeting FCRL isoform(s) may have a completely different mechanism of action from antibodies that bind to and eliminate malignant cells expressing tumor antigens. Antibodies targeting specific FCRL molecules could boost immune recognition of B-cell lymphomas and inhibit tumor growth, so they are worthy of being explored.

The primary objective of this mini-review is to focus on the role of Fc Receptor-Like (FCRL) proteins, particularly FCRL1, in modulating immune recognition and evasion in B-cell lymphomas, with the goal of developing targeted antibody-based immunotherapies. Given that B-cell malignancies often evade immune surveillance through mechanisms that impair antigen recognition [14,15], this review hypothesizes that FCRL1, a predominantly B-cell-expressed molecule, plays a critical role in suppressing T-cell immunosurveillance and immune-mediated tumor clearance. By characterizing the function of FCRL1 in human B-cell lymphomas, the research aims to determine whether targeting this specific FCRL isoform can enhance immune recognition and inhibit tumor growth, offering a more precise therapeutic approach compared to conventional chemotherapy and radiation.

The rationale for this study stems from the limited understanding of FCRL1’s role in human B-cell malignancies, despite evidence suggesting its involvement in B-cell signaling and immune regulation [16,17]. Given the success of antibody therapies, like rituximab, in treating B-cell lymphomas [12,18], the study posits that an antibody targeting FCRL1 may function differently by modulating immune activation rather than simply inducing cell depletion. Since FCRL1 may obstruct CD4^+^ T-cell recognition, its blockade could restore immunosurveillance and improve tumor clearance. Thus, this review seeks to cover the mechanistic role of FCRL1 in immune evasion and evaluate its potential as a novel immunotherapeutic target for B-cell lymphomas, which could lead to more effective and selective treatment strategies. While the importance and role of FCRL1 in B cells and DLBCL are reported, this mini-review highlights the critical role of FCRL1 in B-cell biology and its therapeutic potential for aggressive B-cell malignancies, particularly diffuse large B-cell lymphoma (DLBCL). FCRL1, which contains immunoreceptor tyrosine-based activation motifs (ITAMs), regulates B-cell activation and is overexpressed in malignant B-cells, suggesting its involvement in tumor survival and proliferation. Given its selective expression in lymphoma cells and the limited efficacy of current DLBCL therapies, FCRL1 emerges as a promising immunotherapeutic target. By modulating B-cell signaling and enhancing anti-tumor immunity, FCRL1-directed strategies could address an unmet clinical need in relapsed/refractory disease. This report underscores the rationale for further preclinical and clinical exploration of FCRL1 targeting, offering a novel mechanism-based approach to improve outcomes in DLBCL and other B-cell malignancies.

## 2 B-Cell Lymphomas

Studies suggest that there are two different groups of lymphomas: (a) Hodgkin’s lymphoma (HL), and (b) Non-Hodgkin’s lymphoma (NHL) [7,19]. During class switching and somatic hypermutation, there is a high risk of transforming mutations, so lymphomas commonly stem from germinal center and post-germinal center B-cells. Because of this variance in B-cell development and maturation, when B-cell malignancies arise, there could be a potential influence on immune recognition and tumor clearance. However, the functional interactions of these cells with other components of the immune system remain unclear.

B-cell lymphomas are a type of non-Hodgkin’s lymphoma (NHL) that originates in B-cells. It is the most common type of lymphoma, and about 85% of all NHLs in the United States are related to B-cells [7,20–22]. NHL typically originates in lymphoid tissues but can spread to other organs. NHLs are known to be a heterogeneous group of lymphoproliferative malignancies with varying patterns of behavior and responses to treatment. Common symptoms associated with general lymphomas include painless enlargement of one or more lymph node areas, which are associated with fever, night sweats, and weight loss. While most B-cell lymphomas are NHLs, there are many different subtypes of B-cell NHLs [21,23], such as Burkitt lymphoma (BL), diffuse large B-cell lymphoma (DLBCL), chronic lymphocytic leukemia (CLL), follicular lymphoma (FL), and mantle cell lymphoma (MCL). Prognosis and treatment depend on the subtype and stage of the cancer at diagnosis. The most common and aggressive type of non-Hodgkin’s B-cell lymphoma in Western countries is DLBCL [24–26], and it accounts for about 25% to 30% of all NHLs [27].

DLBCL develops from abnormal B-cells, and when examined under a microscope, the cells appear more spread out (hence the name diffuse) compared to other B-cell malignancies, which remain clumped together [28,29]. It can either develop as a transformation from a less aggressive form of lymphoma or as a first occurrence. Although the disease can occur in childhood, the occurrence of DLBCL likely increases with age, and the majority of patients are over the age of 60 at diagnosis [30]. The most common symptom of DLBCL is swollen lymph nodes in the affected area; these lumps can most often be detected from the surface, but sometimes, they are deep in the body and cannot be felt. Although it is most commonly seen in lymph nodes, about 40% of people develop DLBCL outside of their lymph nodes, known as extranodal disease [31]. Symptoms vary between patients with DLBCL depending on where it is in the body and what tissues are affected. In some cases, patients may even experience flu-like symptoms or weight loss.

There are three main molecular subtypes of DLBCL: activated B-cell-like (ABC) DLBCL, germinal center B-cell-like (GCB) DLBCL, and unclassifiable/type 3 DLBCL [32,33]. ABC DLBCL arises from post-germinal center B-cells (such as plasmablasts) and is typically more aggressive and less responsive to treatment. In contrast, GCB DLBCL typically originates from germinal center B-cells and is more responsive to current treatments. Unclassifiable/type 3 DLBCL has differing molecular characteristics from ABC and GCB types. Type 3 DLBCL typically responds better to treatment than ABC DLBCL, but not as well as GCB DLBCL. Although largely still unknown, studies suggest that FCRL1 expression may vary between the subtypes [16,17,34]. FCRL1 is seen to be more highly expressed in GCB DLBCL compared to ABC DLBCL. This difference in expression patterns may contribute to more subtype-specific immunotherapies and improve treatment outcomes for patients.

## 3 B-Cell Lymphomas and Current Therapies

The current first-line treatment for DLBCL is an anti-CD20 monoclonal antibody, known as Rituximab, used in combination with harsh chemotherapy drugs; these drug combinations, along with Rituximab are known as R-CHOP (R = Rituximab, C = Cyclophosphamide, H = Doxorubicin Hydrochloride/Hydroxy-daunomycin, O = Vincristine Sulfate/Oncovin, P = Prednisone) [24,35,36]. The R-CHOP combination of immunotherapy and chemotherapy drugs may also be used in combination with radiation therapy if the DLBCL is caught in an early stage [36,37]. R-CHOP has been shown to have high remission rates, but there are also major downfalls to using this treatment. One major downfall is that CD20 is present on both malignant and healthy B-cells, so targeting this antigen causes the depletion of healthy cells as well as cancerous cells. This can weaken the patient’s immune system and leave them more susceptible to other infections. Other side effects of using the R-CHOP treatment range from short-term problems such as nausea and hair loss to long-term problems such as heart problems and relapse.

Epcoritamab and Glofitamab may also be used as immunotherapy for treating relapsed or refractory DLBCL [38]; they are bispecific T-cell engagers (BiTEs) that attach T-cells (on the CD3 antigen) and lymphoma cells (on the CD20 antigen) to each other, so the T-cells can remain within close proximity to the lymphoma cells to fight [39]. Many patients benefit from CD20-targeted therapies because of its high expression on tumor cells, but those who relapse after treatment may consider a new CD19-targeted therapy (e.g., Loncastuximab tesirine) because the CD19 antigen has been found to be expressed in most B-cell malignancies [40]. The use of CAR-T cell therapies that target the CD19 antigen, such as axicabtagene ciloleucel, tisagenlecleucel, or lisocabtagene maraleucel, has also been shown to benefit a patient who has relapsed. Similar to R-CHOP, these treatments come with their own downfalls. Some of the biggest downfalls of using BiTEs or CAR-T therapies are the possibilities of antigen escape, off-tumor toxicity, and incomplete tumor clearance.

In addition to R-CHOP, CD19, and CD20 targeted therapies, many other combinations of chemotherapy drugs, radiation therapies, stem cell therapies, and immunotherapies are used to treat DLBCL, depending on the patient’s circumstances. Each of these therapies comes with its own set of limitations, which highlights the need for additional biomarkers that can be more selectively expressed on malignant cells.

## 4 Fc Receptors and Fc Receptor-Like (FCRL) Proteins

An Fc Receptor (FcR) is a protein that contributes to the immune system and is found on the cell surface of many different types of immune cells [41]. FcRs are thought to be key components of currently used antibody-mediated therapies for lymphoma and other cancers. These receptors recognize and bind to the Fc region of an antibody, opposite to the antigen-binding sites, to stimulate an immune response. Fc Receptor Like (FCRL) proteins are a subgroup of immunoglobulin superfamily (IgSF) lymphocyte receptors that are clustered on the long arm of chromosome 1. While some members are found in both B and T cells, FCRL expression is mostly seen in B-cells, including malignant B-cells [42–46].

Members of the human FCRL family consist of six type-1 transmembrane glycoproteins (FCRL1–6) that are attached to the surface of the cell [47,48], as well as two intracellular proteins (FCRLA, FCRLB) that are able to be secreted. Each of the FCRL1–6 proteins expresses immunoglobulin-like (Ig-like) domains on the surface of the cells (extracellular regions/N-terminus), immunoreceptor tyrosine-based inhibiting motif (ITIM) and/or activating motif (ITAM)—like sequences in their cytoplasmic tails (C-terminus) [48], suggesting that they transmit activatory and/or inhibitory signals to B-cells after ligation [43,49]. These FCRL proteins have shown the potential to regulate human B-cell responses. FCRL2–6 have each been found to have at least one ITIM-like motif, and FCRL2, FCRL3, and FCRL5 have both ITIM and ITAM-like sequences, suggesting the capability of dual modulation [48,50]. FCRL1 and FCRL5 differ from the rest of the family members because they contain two of the same sequences in their cytoplasmic tails. FCRL5 contains two ITIM-like motifs and is the largest FCRL family member with nine extracellular Ig-like domains, whereas FCRL1 contains two ITAM-like motifs, suggesting that it is a co-activation receptor [51,52]. Studies also found FCRL1 and FCRL5 to be expressed more than the other members of the human FCRL family in B-cells [53,54], indicating that these proteins may be potential targets for antibody-based treatments of B-cell malignancies such as leukemias and lymphomas. Further research has been conducted on the FCRL family members, indicating that ITIM-like motifs (present in FCRL2–6) have adversely affected B-cell antigen activation [55], drawing even more attention to FCRL1. In addition to its two ITAM-like motifs, FCRL1 contains three extracellular Ig-like domains and no ITIM-like motifs [51,52]. FCRL1 is also the only protein in the family with a negatively charged glutamic acid residue in its transmembrane domain, whereas FCRL2–6 are hydrophobic and have no charge. The restricted distribution of FCRL1 among B-lineage cells, its tightly regulated expression patterns during germinal center B-cell development, and its role in promoting cellular and humoral immune responses suggest its involvement in B-cell transformation [17]. These distinct differences between FCRL1 and the rest of the FCRL family give reason for FCRL1 to be a key player in B-cell lymphoma, and the main focus of the FCRL family as a target.

## 5 FCRL1 and B-Cell Lymphoma

**Figure 1:**
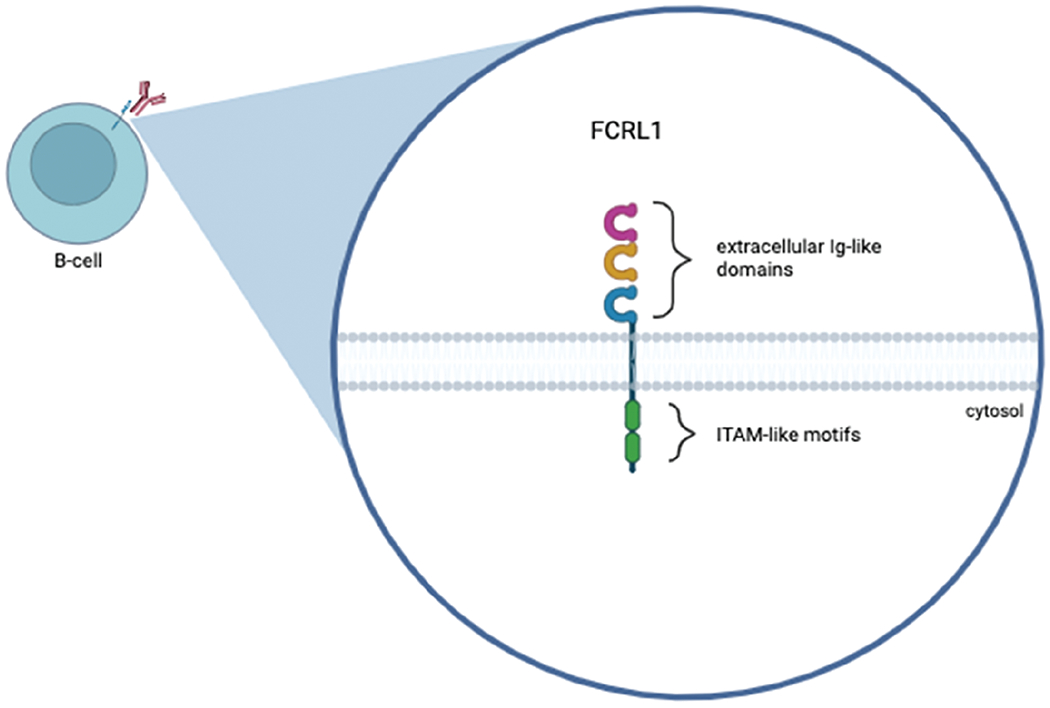
Graphical representation of the human FCRL1 protein. FCRL1 is a cell-surface membrane protein that belongs to the FCRL family. The figure shows three Ig-like extracellular domains, a transmembrane domain, and a cytoplasmic domain with two immunoreceptor-tyrosine activation motifs. This figure was created using Biorender software (version 201)

FCRL1 is a protein-coding gene. The protein it encodes features three extracellular C2-type immunoglobulin domains, a transmembrane domain, and a cytoplasmic tail containing two ITAMs (Fig. 1). The idea of FCRL1 as a potential target for antibody-based treatments of B-cell malignancies has already begun to be explored. Du et al. found 83% of patients (34/41) with a type of B-cell lymphoma, such as CLL, FL, MCL, or hairy cell leukemia (HCL), to express FCRL1 proteins on their malignant cells [47]. By using FCRL1-specific mAbs, this group also confirmed the frequent expression of FCRL1 on CLL, FL, and some other B-cell malignancies. Although Du et al. do not explicitly talk about DLBCL within patients, cell lines REC1, OCI-Ly7, and SU-DHL-6, which are associated with DLBCL, were also included in the study. The REC1 cell line showed the highest expression of FCRL1 out of all cell lines used in the study [47], proving the benefit of including DLBCL when looking at FCRL1 as a potential target for antibody-based treatments. Several other studies also indicated differential expression levels of FCRL1 transcripts by primary FL, CLL, MCL, and DLBCL [56,57], CLL, and in some cases BL, showing the highest expression. However, DLBCL is the most aggressive type of B-cell NHL, and the role and mechanisms of FCRL1 in DLBCL remain unclear. Thus, this mini-review focuses on the role of FCRL1 in B-cell NHLs, particularly DLBCL.

### 5.1 FCRL1 Expression and Signaling

FCRL1 expression has also been shown to differ between the ABC (activated B-cell-like) and GCB (germinal center B-cell-like) subtypes of DLBCL [16,17], reflecting the biological heterogeneity of these subgroups. Studies indicate that FCRL1 is more highly expressed in the GCB subtype compared to the ABC subtype [17], consistent with its association with germinal center B-cells, where FCRL1 is commonly upregulated during B-cell maturation. This differential expression may contribute to the distinct clinical behaviors and therapeutic responses observed between ABC and GCB DLBCL, as FCRL1 has been implicated in modulating BCR signaling and immune regulation. The stronger expression in GCB DLBCL suggests a potential role in germinal center-derived lymphomagenesis or tumor microenvironment interactions, whereas its lower expression in ABC DLBCL may reflect the more aggressive, post-germinal center nature of this subtype. Further research is needed to correlate expression levels with disease progression and therapeutic response.

While FCRL1 is known to modulate B-cell activation and proliferation through its immunoreceptor ITAMs, the specific downstream signaling pathways it engages in DLBCL remain poorly defined. Unlike canonical ITAM-containing receptors like the BCR, where the signaling cascade through SYK, BTK, and PLC*γ*2 is well characterized, the extent to which FCRL1 engages similar or distinct effectors remains unclear. Furthermore, it is unknown whether FCRL1 signaling functions independently or cooperatively with chronic active BCR signaling, which is a key driver in the ABC subtype of DLBCL.

Less information is available on whether FCRL1 amplifies BCR signals, modulates threshold sensitivity to antigen, or provides survival cues under tonic signaling conditions. However, it remains possible that FCRL1 acts as a co-receptor, influencing BCR microcluster formation, immunological synapse stability, or downstream kinase activation. Additionally, the interplay between FCRL1 and other regulators of B-cell fate (e.g., PI3K/AKT, NF-κB, or MAPK pathways) has not been systematically mapped in the DLBCL context. Addressing this gap requires further investigation on dissecting FCRL1-associated signalosomes using loss-of-function studies in FCRL1-high DLBCL models. A deeper mechanistic understanding of how FCRL1 drives or supports lymphomagenesis could provide a rationale for targeting this molecule directly or in combination with established pathway inhibitors.

### 5.2 FCRL1 and Lymphoma Immunotherapy

Because of FCRL1’s expression on malignant B-cells and emerging role in modulating B-cell receptor (BCR) signaling, it has become a promising candidate for antibody-based interventions. Unlike current therapies that target CD19 or CD20, FCRL1 may offer a distinct therapeutic mechanism by modulating intracellular activation pathways that would be critical for the survival and proliferation of B-cell lymphomas. Recent studies have begun to explore how FCRL1 influences tumor behavior and responses to treatments, which may lay the groundwork for future therapy options.

Zhao et al. investigated the functional role of FCRL1 by generating FCRL1-deficient CH27 (CH27-FCRL1-KO) cells using the CRISPR-Cas9 approach with a single-guide RNA (sgRNA) [58]; these cells targeted the third exon of the FCRL1 gene. They were able to compare the strength of B-cell receptor signaling by measuring the amount of BCRs that accumulated at synapses. Quantitative analysis revealed that, upon BCR cross-linking alone, CH27-FCRL1-KO cells showed markedly reduced accumulation of BCRs in the immunological synapse when compared to the CH27 wild-type cells. Because CH27 cells are a mouse B-cell lymphoma cell line, the results can be considered a topic needing more exploration for human B-cell lymphoma cell lines. The findings from this study indicate FCRL1 plays an important role in promoting tumor growth. Furthermore, Zhao et al. examined the distribution of FCRL1 molecules during BCR engagement to understand why deficiency in FCRL1 proteins leads to impaired BCR-mediated activation of B-cells due to the lack of FCRL1 ligation [58]. The analysis of the unusual distribution of both BCR and FCRL1 proteins within the immunological synapse of B-cells suggested that FCRL1 ligation could strongly induce the aggregation of FCRL1 molecules, but not BCRs. Overall, this study provided evidence that the expression of FCRL1 plays a positive regulatory function in B-cell activation and antibody responses, suggesting that more in-depth studies are required to explore its potential antibody-based therapeutic use in patients with malignant B-cell lymphomas.

**Figure 2:**
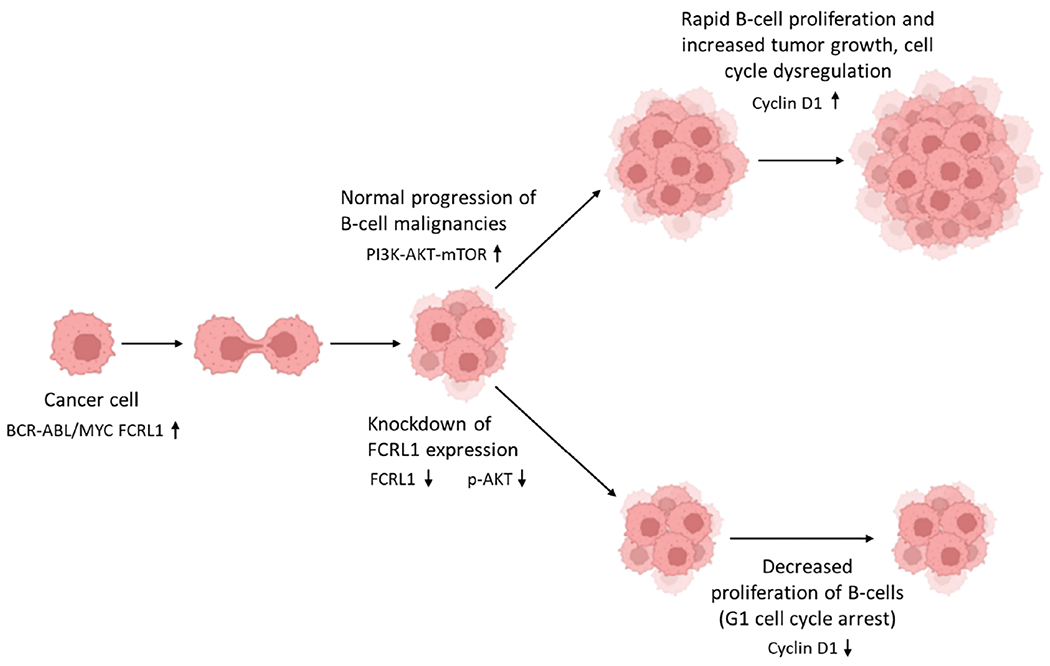
FCRL1 knockdown impairs B-cell lymphoma growth by disrupting pro-survival signaling. In B-cell malignancies driven by BCR-ABL and MYC oncogenic signaling, FCRL1 overexpression sustains cancer cell proliferation via activation of the PI3K-AKT-mTOR pathway, leading to elevated Cyclin D1 expression and unchecked tumor growth. Following FCRL1 knockdown, malignant B-cells exhibit reduced PI3K-AKT-mTOR signaling (low pAKT), resulting in downregulation of Cyclin D1 and subsequent G1 cell cycle arrest. This suppression of proliferative drivers correlates with significantly reduced tumor growth, highlighting FCRL1 as a potential therapeutic target in B-cell malignancies. This figure was created using Biorender software (version 201)

Yousefi et al. expanded the study of FCRL1 to include DLBCL cells and compared the levels to healthy controls [52]. Results from this study indicated a notable overexpression of FCRL1 on the neoplastic B-cells of DLBCL patients compared to the non-malignant B-cells of healthy controls in both protein (*p* < 0.0001) and mRNA (*p* < 0.001) [52]. When analyzing the ablation of FCRL1 expression, there was reduced B-cell proliferation within DLBCL, as illustrated in Fig. 2. Since upregulation of FCRL1 proteins has the potential to amplify B-cell activation, and abnormal expression of this protein has been reported in DLBCL cell lines as well as BL, CLL, MCL, HCL, and FL patients [52,53], FCRL1 could be involved in the pathogenesis of B-cell malignancies, specifically DLBCL. DLBCL was especially emphasized in the study by Yousefi et al. because FCRL1 was able to be detected in 80.7% of DLBCL patients [52], highlighting the importance of FCRL1 as a marker for DLBCL and its importance in immunotherapy. Quantitative studies reveal that FCRL1 is expressed at significantly higher levels in malignant B-cells compared to healthy B-cells [17,34,47,52]. In normal B-cell populations, FCRL1 shows restricted and transient expression, primarily during early B-cell development, with minimal to undetectable levels in mature naïve or memory B-cells. In contrast, malignant B-cells such as those in CLL, DLBCL, MCL, exhibit pronounced FCRL1 upregulation, often confirmed by qPCR, flow cytometry, and RNA sequencing. For instance, CLL cells demonstrate 5- to 10-fold higher FCRL1 mRNA levels than normal B-cells, with protein expression similarly elevated. This differential expression suggests FCRL1 as a potential biomarker for B-cell malignancies and implies its functional role in promoting survival, proliferation, or immune evasion in transformed B-cells.

## 6 Current Standards, Synergy with Newer Immunotherapies, Advantages and Future Directions

FCRL1 represents a novel target that could address key limitations of existing therapies like R-CHOP and CD19/CD20-directed agents [16,52]. While R-CHOP combines chemotherapy with anti-CD20 (rituximab), resistance often arises from CD20 loss or TP53 mutations [59,60]. FCRL1, however, is expressed on malignant B cells (e.g., CLL, NHL) while largely absent on plasma cells, potentially sparing humoral immunity [16,17]. This makes it a promising alternative for CD20-low or CD19-negative relapses, where current standards fail. Additionally, FCRL1’s restricted expression on malignant cells may reduce off-target toxicity compared to broader B-cell depletion strategies.

FCRL1 could complement next-generation immunotherapies by overcoming antigen escape mechanisms. BiTEs (e.g., CD20 × CD3 or CD19 × CD3) and CAR-T therapies are highly effective but limited by CD19/CD20 downregulation in relapsed disease [61–63]. An FCRL1-targeted approach, whether through bispecifics (FCRL1 × CD3), CAR-T cells, or antibody-drug conjugates (ADCs), could provide a salvage option for such cases. Preclinical data suggest FCRL1 CAR-T efficacy, positioning it as a sequential therapy post-CD19 CAR-T failure or even as part of dual-targeted strategies to prevent relapse [64–66]. Although still early, these approaches offer hope for more precise and effective treatments for B-cell lymphomas.

Researchers are also considering combining FCRL1 therapies with other treatments, such as checkpoint inhibitors or drugs that block cancer survival signals. Unlike CD19/CD20, FCRL1’s expression profile may offer a more tumor-selective window, enabling mechanisms like ADCs or immune redirection, with fewer side effects. Challenges remain, including defining optimal combinations (e.g., with R-CHOP or checkpoint inhibitors) and monitoring for FCRL1 loss as a resistance mechanism. Clinical trials will be crucial to validate whether FCRL1 targeting can fill unmet needs in refractory B-cell malignancies, particularly where current standards and newer immunotherapies fall short.

## 7 Conclusions

FCRL1 has emerged as a highly promising therapeutic target in B-cell malignancies, particularly due to its selective overexpression in aggressive cancers like DLBCL and its potential role in driving tumor progression. Its unique expression pattern, abundant in malignant B-cells but largely absent in normal plasma cells, positions it as an ideal candidate for precision immunotherapy, minimizing off-target toxicity and preserving humoral immunity. This specificity is especially valuable in addressing CD19/CD20-negative relapses, a major clinical challenge where current therapies like R-CHOP or CD19-directed CAR-T cells often fail. The development of FCRL1-targeted modalities, including bispecific antibodies, next-generation CAR-T cells, and ADCs, could circumvent antigen escape mechanisms that limit the durability of existing treatments.

Beyond its therapeutic potential, FCRL1 may also serve as a diagnostic or prognostic biomarker, enabling earlier detection and risk stratification, a critical advancement for elderly patients who constitute the majority of DLBCL cases and face poorer outcomes. However, gaps remain in understanding FCRL1’s biological functions in normal B-cell development and its precise oncogenic mechanisms. Future research must prioritize translational studies with diverse patient cohorts, including underrepresented demographics, varied disease stages, and genetic backgrounds, to ensure broad applicability. In addition, investigating FCRL1’s interplay with tumor microenvironmental factors and resistance pathways (e.g., FCRL1 loss or downregulation) will be essential for optimizing therapeutic strategies.

Clinically, the greatest promise of FCRL1 lies in addressing unmet needs in refractory or relapsed disease, where salvage options are limited. Early-phase trials should explore combination approaches, such as pairing FCRL1-directed therapies with immune checkpoint inhibitors or targeted agents, to maximize efficacy and overcome resistance. While challenges like on-target/off-tumor effects or adaptive immune evasion require careful monitoring, preclinical data robustly support FCRL1’s candidacy for rapid clinical translation. Ultimately, successful targeting of FCRL1 could redefine treatment paradigms for high-risk B-cell malignancies, offering a much-needed lifeline to patients with otherwise dismal prognoses.

## Data Availability

The datasets generated or analyzed during the current study are available from the corresponding author on reasonable request.
